# Case Report: Severe depression and suicidality in a young patient with RYR1-related congenital myopathy

**DOI:** 10.3389/fpsyt.2026.1970738

**Published:** 2026-10-27

**Authors:** Ştefan Adrian Radu, Octavian Vasiliu, Corina Tudor

**Affiliations:** 1 Department of Psychiatry, Dr. Carol Davila University Emergency Central Military Hospital, Bucharest, Romania; 2 Discipline of Psychiatry II, Department of Clinical Neurosciences, Carol Davila University of Medicine and Pharmacy, Bucharest, Romania

**Keywords:** case report, congenital myopathies, major depressive disorder, obsessive compulsive disorder, psychiatric comorbidity, RYR1-related myopathy, suicidal ideation

## Abstract

**Background:**

RYR1-related congenital myopathies (RYR1-CMYO) are rare inherited neuromuscular disorders characterized by considerable clinical and histopathological heterogeneity. Although chronic physical disability may be associated with substantial psychological burden, psychiatric manifestations in patients diagnosed with RYR1-related disorders remain insufficiently characterized.

**Case presentation:**

We report the case of an 18-year-old man with a longstanding RYR1-related congenital myopathy with an autosomal dominant family pattern, who was admitted for a severe depressive episode with active suicidal ideation and a suicide plan. The patient also met diagnostic criteria for obsessive-compulsive disorder and retrospectively reported psychotic symptoms of uncertain timing, duration, and diagnostic significance. No active psychotic symptoms were identified at the documented examination. The familial RYR1 p.Leu4579Arg missense variant had previously been identified in both the patient and his clinically affected mother, supporting segregation with the neuromuscular phenotype. However, patient-specific muscle histopathology was unavailable. A particularly salient psychosocial feature was marked resentment toward his mother, whom he perceived as responsible for transmitting the disorder, in the context of longstanding physical disability, reduced autonomy, and intrafamilial conflict. An individualized multimodal treatment approach combining pharmacotherapy, described family-oriented work, suicide-risk management, and multidisciplinary follow-up was associated with partial improvement in depressive symptoms and reduction of active suicidal ideation during follow-up. The contributions of individual interventions could not be separated.

**Conclusion:**

This case illustrates the psychiatric and psychosocial burden of living with an inherited disabling neuromuscular disease in a young adult. Although a direct pathophysiological relationship between RYR1 dysfunction and major depressive disorder or an RYR1-specific psychiatric phenotype cannot be inferred, chronic disability, impaired autonomy, awareness of genetic transmission, and associated family dynamics may contribute to psychological distress. The case highlights the importance of systematic assessment of depression and suicide risk in patients with rare disabling neuromuscular disorders and supports the integration of psychiatric care, psychological support, genetic counseling, and family-oriented interventions within multidisciplinary management.

## 1 Introduction

Congenital myopathies (CMs) are a heterogeneous group of genetic muscle disorders, typically presenting with hypotonia and weakness (1). RYR1-related disease encompasses several clinical and histopathological phenotypes (2). Contemporary nomenclature integrates the gene and inheritance pattern with histopathology when available (3, 4).

The impact of inherited neuromuscular disease extends to daily functioning, social participation, and family life (5). Studies of RYR1-related disorders describe impaired quality of life and concerns reported by patients and caregivers (6, 7). This report describes severe depression with suicidal ideation and comorbid obsessive-compulsive disorder in a young person with RYR1-related congenital myopathy. Its principal contribution is to illustrate the psychiatric and psychosocial burden associated with disability, reduced independence, family relationships, and the patient’s understanding of genetic transmission, rather than to establish an RYR1-specific psychiatric phenotype.

Genetic counseling can address inheritance, uncertainty about severity, and reproductive choices. A parent carrying a heterozygous autosomal dominant disease-causing variant has a 50% probability of transmitting that variant in each pregnancy; this does not predict disease severity or psychiatric outcomes. Targeted prenatal testing may be considered following specialist evaluation of the familial variant and available testing options (8). Counseling should support informed decisions, including the decision not to undergo testing. Studies in Huntington disease illustrate the complexity of reproductive choices and perceived responsibility within families (9, 10). This comparison concerns the psychosocial context of inherited disease and does not establish a shared psychiatric mechanism.

The report was constructed in accordance with the CARE (CAse REport) guidelines (Supplementary material 1) (11). Clinical information was retrospectively collected from the patient’s available medical records, including medical history, clinical and neurological examinations, laboratory and imaging investigations, historical accounts of maternal histopathology, genetic testing, therapeutic interventions, and follow-up data, where available. Relevant information regarding family history and genetically affected relatives was also reviewed when pertinent to the diagnosis and interpretation of the case. The clinical course, diagnostic work-up, and management were organized chronologically. The study was conducted in accordance with the principles of the Declaration of Helsinki and was approved by the Local Ethical Committee of the “Dr. Carol Davila” University Emergency Military Central Hospital (no. 879/2026). Written informed consent was obtained from the patient for the publication of clinical and genetic data.

## 2 Case report

An 18-year-old male patient with a history of RYR1-related congenital myopathy presents with psychiatric symptoms characterized by depressed mood, generalized anxiety with paroxysmal episodes, internal psychological tension, hyperphagia with weight gain, social withdrawal, and recurrent, active suicidal ideation associated with a plan for self-harm, in the context of longstanding neuromuscular disability.

The patient presented with a significant perinatal history, including birth at 38 weeks’ gestation, neonatal respiratory distress syndrome, marked hypotonia (“floppy infant”), and indirect hyperbilirubinemia. The course of the disease was chronic, with longstanding motor impairment, requiring orthopedic interventions (bilateral Achilles tendon tenotomy in 2012) and medical rehabilitation measures. Currently, the patient presents with predominantly proximal motor deficits in the lower limbs, gait disturbances requiring a cane outside the home, and difficulties performing daily activities.

The diagnosis of CM in this patient was established early and genetically confirmed in July 2009 at the Institute of Myology in Paris by identifying the familial RYR1 p.Leu4579Arg variant, present in both the patient and his clinically affected mother. The diagnosis is described as RYR1-related congenital myopathy with an autosomal dominant family pattern; patient-specific histopathological confirmation is unavailable (3). His mother underwent two biopsies performed at 4 and 6 years of age, described as showing central core disease in the July 2009 specialist account, whereas the August 2009 letter used the term “centronuclear myopathy”; she had a slowly progressive childhood-onset myopathy. Unfortunately, the mother did not benefit from a genetic/molecular diagnosis until 2009, so she did not have confirmation of the disease etiology. The original laboratory report considered several findings supportive of a causal role of the variant in the familial myopathy but noted that functional confirmation was lacking.

The familial variant was identified after the patient’s birth in 2007, and information about preconception or prenatal genetic counseling and testing was unavailable. The patient’s attribution of responsibility to his mother represents his reported illness perception and does not establish what the parents knew or which reproductive options were available to them. The specialist letter of 5 August 2009 recommended formal genetic counseling in Romania or during a subsequent visit to the Institute of Myology, but the available documents do not confirm whether this consultation took place.

A systematic psychiatric history of the maternal and paternal family, including the patient’s mother, was unavailable, so that familial vulnerability to mood disorders, OCD, psychotic disorders, substance-use disorders, and suicidal behavior could not be assessed. In this context, it is relevant to note that missing documentation does not establish a negative family psychiatric history.

Functional assessments reveal a Modified Katz Activities of Daily Living (ADL) score of 9/10, a Barthel score of 85/100, and bilateral muscle strength of grade 4/5. The neurological examination reveals a proximal motor deficit, inability to perform maneuvers such as standing from a seated position without support and squatting, and gait disturbances with postural asymmetry. Somatic comorbidities include thoracic kyphosis, lumbar hyperlordosis, “S”-shaped scoliosis, and bilateral flatfoot with valgus. Also, class I obesity (body weight 90 kg; BMI = 31.5 kg/m^2^) according to the 2013 AHA/ACC/TOS criteria (12).

From a psychiatric perspective, the patient has a history of anxiety and depressive symptoms, intermittent suicidal ideation retrospectively dated to 2019, as well as obsessive-compulsive features and retrospectively reported psychotic symptoms. The date 2019 identifies the earliest reported suicidal ideation but does not establish the onset of a syndromal depressive episode. Psychological evaluations (ASEBA - Achenbach System of Empirically Based Assessment and Conners-3) revealed significant affective and anxiety symptoms, with no clinical criteria for ADHD.

In 2025, the patient was diagnosed with a severe major depressive episode and obsessive-compulsive disorder, according to DSM-5-TR criteria (13), and was placed on psychotropic medication (sertraline, aripiprazole, gabapentin), with a partially favorable therapeutic response.

During the same period, the patient was in relatively good general condition, as self-reported and admitted by his mother and treating physician. Physical examination revealed deformities of the musculoskeletal system (thoracic kyphosis, lumbar hyperlordosis, scoliosis), as well as bilateral flat feet with valgus deformity.

Neurological examination revealed a predominantly proximal motor deficit in the lower extremities, with decreased muscle strength (grade 4/5 bilaterally), inability to perform functional maneuvers (standing up from a seated position without support, squatting), gait disturbances with postural asymmetry, and the need for assistive devices for mobility. Deep tendon reflexes were absent throughout, and coordination is partially limited by the motor deficit.

Electromyography (EMG) findings were consistent with mild chronic myopathy, with predominant myogenic features. Brain CT scan did not reveal significant pathological changes.

The detailed psychiatric evaluation revealed the patient was conscious, cooperative, and oriented in time and space, with intact self-awareness and awareness of others. Appropriate, neat attire, with reduced facial expressions and gestures. Eye contact was easy to initiate but difficult to maintain. Spontaneous speech present, focused on medical and relational issues. Mild attentional deficits and mild hypomnesia were detected. Coherent thinking, with preserved logical associations, intact capacity for abstraction and synthesis. Depressive mood, anhedonia, internal psychological tension, irritability, low frustration tolerance, and social withdrawal. Thoughts of worthlessness, helplessness, and guilt in the context of the somatic illness and interpersonal relationships. Marked ambivalence toward his mother is noticeable, stemming from the attribution of responsibility for the transmission of the illness. Recurrent, active suicidal ideation, associated with a suicide plan, was reported. The retrospective psychiatric history included persecutory and auditory experiences reported by the patient and supported by his mother’s account, as well as obsessive-compulsive phenomena (intrusive thoughts and ritualistic behaviors). The dates, duration, relationship to mood symptoms, and diagnostic significance of the earlier psychotic symptoms could not be established. At the time of examination, no active psychotic symptoms were evident. Also noticed was hyperphagia with weight gain. Hypobulia, overall reduced activity, a tendency toward isolation, and a good level of insight were present. The patient was adherent to the recommended treatment.

The psychiatric diagnoses, based on a review of the patient’s medical history, clinical history, physical and neurological examination, psychiatric evaluation, and response to treatment, are as follows (DSM-5-TR): Major depressive disorder (MDD), current episode severe, without current psychotic features; Obsessive-compulsive disorder (OCD); RYR1-related congenital myopathy with an autosomal dominant family pattern (familial RYR1 p.Leu4579Arg variant). Arguments for the severe major depressive episode were persistent depressed mood, marked anhedonia, fatigue and decreased activity, decreased self-esteem and feelings of worthlessness, feelings of guilt (centered on social rejection and illness), social withdrawal, low frustration tolerance, concentration difficulties, active suicidal ideation with a plan, and changes in appetite (hyperphagia with weight gain). The obsessive-compulsive disorder diagnosis was supported by recurrent intrusive thoughts (scenes with anxiety-provoking content), compulsive neutralizing behaviors (religious and behavioral rituals), present insight, and egodystonic character of the intrusive thoughts. The neurological diagnosis was RYR1-related congenital myopathy, without patient-specific histopathological confirmation, with proximal motor deficit, assisted walking, and moderate functional impairment (ADL 9/10, Barthel 85/100). The data collected on patient history are insufficient to establish separate major depressive episodes and intervening remission, so a recurrent course is not asserted.

The psychiatric *differential diagnosis* included bipolar disorder, schizophrenia spectrum disorders, depressive disorder due to another medical condition, adjustment disorder, and generalized anxiety disorder. (1) Bipolar disorder was considered unlikely because no previous manic or hypomanic episodes could be identified in the available clinical history. (2) Schizophrenia spectrum disorders were also considered in view of the retrospectively reported persecutory and auditory experiences; however, the available longitudinal information did not document persistent psychotic symptoms or a period of psychosis occurring independently of prominent mood symptoms, and no active psychotic manifestations were identified during the current assessment; still, given the limited retrospective information regarding the earlier reported psychotic symptoms, their precise diagnostic significance remains uncertain and warrants longitudinal monitoring. (3) Depressive disorder due to another medical condition was considered because of the underlying RYR1-related congenital myopathy; however, the available evidence does not establish a direct pathophysiological relationship between RYR1 dysfunction and depressive illness; the depressive syndrome was therefore conceptualized as comorbid major depressive disorder occurring in the context of a chronic disabling medical condition rather than as a direct physiological consequence of the myopathy. (4) Adjustment disorder was considered less appropriate because the patient fulfilled criteria for major depressive disorder, including a pervasive depressive syndrome with marked anhedonia, feelings of worthlessness and guilt, neurovegetative changes, functional impairment, and recurrent suicidal ideation with a plan. (5) Although clinically significant anxiety symptoms were present, the available assessment did not support an independent generalized anxiety disorder, and these manifestations were interpreted in the context of the depressive syndrome and associated psychosocial distress.

The treatment of this patient had as main therapeutic goals the remission of depressive symptoms, reduction of suicide risk, control of obsessive-compulsive symptoms, emotional and behavioral stabilization, improvement of overall functioning, increased treatment adherence, and social and family reintegration.

The treatment regimen used included sertraline 100 mg/day (starting from 25 mg daily, with gradual increments) for depression and obsessive-compulsive disorder (14–17). An atypical antipsychotic, i.e., aripiprazole, was added due to residual significant symptoms and titrated to 10 mg bid (18, 19). Gabapentin was also used at a total daily dose of 900 mg, as confirmed by careful review of the medical charts, to reduce anxiety and psychic tension (20, 21). Also, lamotrigine 100 mg/day was added to address affective instability (22, 23).

Although lamotrigine and gabapentin are not routinely administered for MDD or OCD, they were considered in this case as individualized off-label add-ons that were introduced for controlling psychiatric symptoms (24). These additions were not based on evidence of efficacy in RYR1-related myopathy; treatment was guided by established recommendations for the individual psychiatric disorders and adapted to the patient’s clinical context.

**Tables 1**, **2** record sertraline with gabapentin in January 2025, aripiprazole augmentation in February 2025, and a regimen including lamotrigine by January 2026. The available documentation does not establish the exact date when gabapentin reached 900 mg/day.

**Table 1:** Chronological clinical course of the patient.

Date	Clinical event	Specialty	Relevant details
2007	Birth	Neonatology	Born at 38 weeks of gestation, 2200 g, hypotonic syndrome (“floppy infant”), respiratory distress
2009	Genetic testing	Neurology	Identification of familial RYR1 variant (p.Leu4579Arg)
2012	Surgery	Orthopedics	Bilateral Achilles tendon tenotomy + immobilization + orthoses
2019	Earliest reported suicidal thoughts	Psychiatry	Suicidal thoughts retrospectively dated to 2019; onset of a syndromal depressive episode not established
January 2025	Psychological evaluation	Psychology	ASEBA, Conners-3: clinical scores (affect, anxiety, somatization); parent–patient discrepancy
January 2025	Psychiatric consultation	Psychiatry	Diagnosis: OCD and severe MDE; initiation of treatment (sertraline + gabapentin); favourable evolution for 3 weeks
February 2025	Psychiatric deterioration	Psychiatry	Recurrent suicidal thoughts that triggered the addition of aripiprazole
March 2025	Revaluation	Psychiatry	Persistence of symptoms that required treatment adjustment, with a gradual increase in aripiprazole and gabapentin dosage
April 2025	Rehabilitation consultation	Neurology	Myopathy + deformities (kyphosis, scoliosis, flat feet, hallux valgus)
April 2025	Cranial CT scan	Psychiatry	No structural abnormalities detected
May 2025	EMG	Neurology	Mild chronic myopathy (myogenic pattern)
May 2025	Neurological evaluation	Neurology	Proximal motor deficit, walking with a cane, inability to stand up without support
May 2025	Functional assessment	Neurology	ADL 9/10, Barthel 85/100, muscle strength 4/5
May 2025	Psychiatric evaluation	Psychiatry	GAF = 55 (moderate impairment), persistent symptoms
Early January 2026	Revaluation	Psychiatry	Treatment: sertraline 100 mg, aripiprazole, gabapentin 900 mg/day, lamotrigine
Last known visit (Late January 2026)	Assessment of clinical progress	Psychiatry	Partially favorable response; emotional vulnerability persists

Dates indicate when findings were recorded or retrospectively reported, not necessarily their onset. Comparable serial motor measurements and dates of specific functional losses were unavailable; therefore, the temporal relationship between physical deterioration and psychiatric worsening could not be established. ADL, Modified Katz Activities of Daily Living; ASEBA, Achenbach System of Empirically Based Assessment; CT, computed tomography; EMG, electromyography; GAF, Global Assessment of Functioning Scale; MDE, major depressive episode; OCD, obsessive-compulsive disorder.

**Table 2:** Evolution of psychiatric treatment.

Date	Medication	Dosage	Changes	Clinical judgment
January 2025	Sertraline + gabapentin	Sertraline: gradually increase the dose up to 50 mg/day; Gabapentin: increase the dose up to 100 mg twice daily	Starting treatment	Severe depressive episode + anxiety; SSRI agent selected for its safety profile
February 2025	Sertraline + gabapentin + aripiprazole	Sertraline 50 mg/day; Gabapentin 100 mg twice daily; Aripiprazole, starting at 2.5 mg/day → increase	Antipsychotic introduction	Antidepressant augmentation + monitoring of suicidal ideation
March 2025	Sertraline + gabapentin + aripiprazole	Gabapentin titrated upward (see dose note); aripiprazole increased gradually	Enhanced treatment	Persistence of anxiety and depression symptoms→ optimization needed
		Aripiprazole increased to ~15 mg/day (in divided doses)	Adjustment	Insufficient control of affective symptoms
April 2025	No major changes in clinical status	—	Maintenance	Relative stabilization
May 2025	Sertraline + gabapentin + aripiprazole	Sertraline 50 mg/day; gabapentin (see dose note); aripiprazole ~15 mg/day	Monitoring	GAF = 55 → moderate functional impairment
Early January 2026	Sertraline + Aripiprazole + Gabapentin + Lamotrigine	Sertraline 100 mg/day; Aripiprazole 20 mg/day; Gabapentin 900 mg/day; Lamotrigine 100 mg/day	Introduction of a stabilizer + optimization	Affective instability and persistent symptoms; individualized off-label adjunct
Last known visit (Late January 2026)	Sertraline + aripiprazole + gabapentin + lamotrigine	Sertraline 100 mg/day; Aripiprazole 20 mg/day; Gabapentin 900 mg/day; Lamotrigine 100 mg/day	Partial recovery	Partially favorable response, vulnerability persists

The exact date when the gabapentin 900 mg/day dose was reached is not established based on available documents. Earlier titration steps are retained where recorded. SSRI, selective serotonin reuptake inhibitor; GAF, Global Assessment of Functioning Scale.

The evolution under treatment was a gradual improvement in mood, a reduction in the intensity of suicidal ideation, increased treatment compliance, and maintenance of insight. The response is considered favorable but partial and requires ongoing monitoring. Improvement occurred during combined care, and the contribution of any individual medication or psychological intervention cannot be determined.

Also, non-pharmacological treatment was recommended, mainly cognitive-behavioral therapy (CBT) focusing on restructuring negative cognitions, managing feelings of worthlessness and guilt, and addressing obsessive symptoms (25–27). Supportive therapy was considered a viable alternative to assist the patient in adapting to chronic illness, resolving conflicts with the mother figure, and developing social skills (28, 29). The actual delivery and intensity of such interventions could not be precisely determined based on the available documents.

The clinical account describes family-oriented work addressing illness perceptions, crisis planning, family roles, communication, and the balance between safety monitoring and autonomy. This psychiatric work should not be equated with formal genetic counseling. Session frequency, duration, specific techniques, and outcomes of individual components were unavailable from the medical records or serial interviews.

By compiling all available data, we can reconstruct the course of the somatic and psychiatric illnesses up to the most recent medical evaluation (**Table 1**). Neuromuscular impairment was present from infancy, preceding the earliest reported suicidal ideation in 2019, and psychiatric assessment in 2025 documented severe depression and OCD. Motor and functional assessments in spring 2025 demonstrated proximal weakness and restricted mobility. The available measurements do not establish when these limitations reached that level or whether deterioration in physical function preceded psychiatric worsening. Longstanding disability is therefore part of the clinical context, while the temporal relationship between changes in physical function and psychiatric symptoms remains uncertain.

Under combined psychiatric treatment, the course is partially favorable, with reduced intensity of depressive mood, diminished suicidal ideation, improvement in anxiety, no active psychotic symptoms at the documented examination, and good treatment adherence (**Table 2**). However, some clinical aspects, such as emotional vulnerability, difficulties with psychosocial adaptation, and a moderate functional impairment, persist. The tolerability of the treatment was good, and although adverse events were not assessed using a structured approach (e.g., a validated instrument), they were collected based on interviews at each visit.

The psychiatric prognosis in this case remains guarded due to the multidimensional interaction of biological, psychological, and social factors. As negative prognostic factors, retrospectively reported suicidal thoughts since 2019, a severe depressive episode with active suicidal ideation and a plan, chronic, disabling neurological disease, functional locomotor impairment (partial dependence), difficulties in developing interpersonal relationships (including emotional and sexual ones), persistent family conflict, and comorbid OCD can all be mentioned. However, favorable prognostic factors exist, such as good insight and treatment adherence, positive therapeutic response (even if partial), absence of psychoactive substance use, and continuous medical support.

## 3 Discussion

This case illustrates the psychiatric and psychosocial burden associated with a rare inherited neuromuscular disorder and severe psychiatric morbidity (MDD, OCD) in a young adult affected by an RYR1-related congenital myopathy with an autosomal dominant family pattern. Beyond the burden associated with the neuromuscular disease itself, awareness of its familial transmission and the patient’s attribution of responsibility to his affected mother emerged as particularly relevant psychosocial features. Although the available evidence does not support a direct causal relationship between RYR1 dysfunction and psychiatric disorders, this case highlights the need to consider the interaction between physical disability, psychological adjustment, family dynamics, and genetic disease awareness when assessing psychiatric morbidity in patients with inherited neuromuscular conditions (**Figure 1**). The individual causal contributions of these contextual factors cannot be determined from a single case. The figure’s calcium-signaling component draws on general neuromuscular research (30–32).

**Figure 1:**
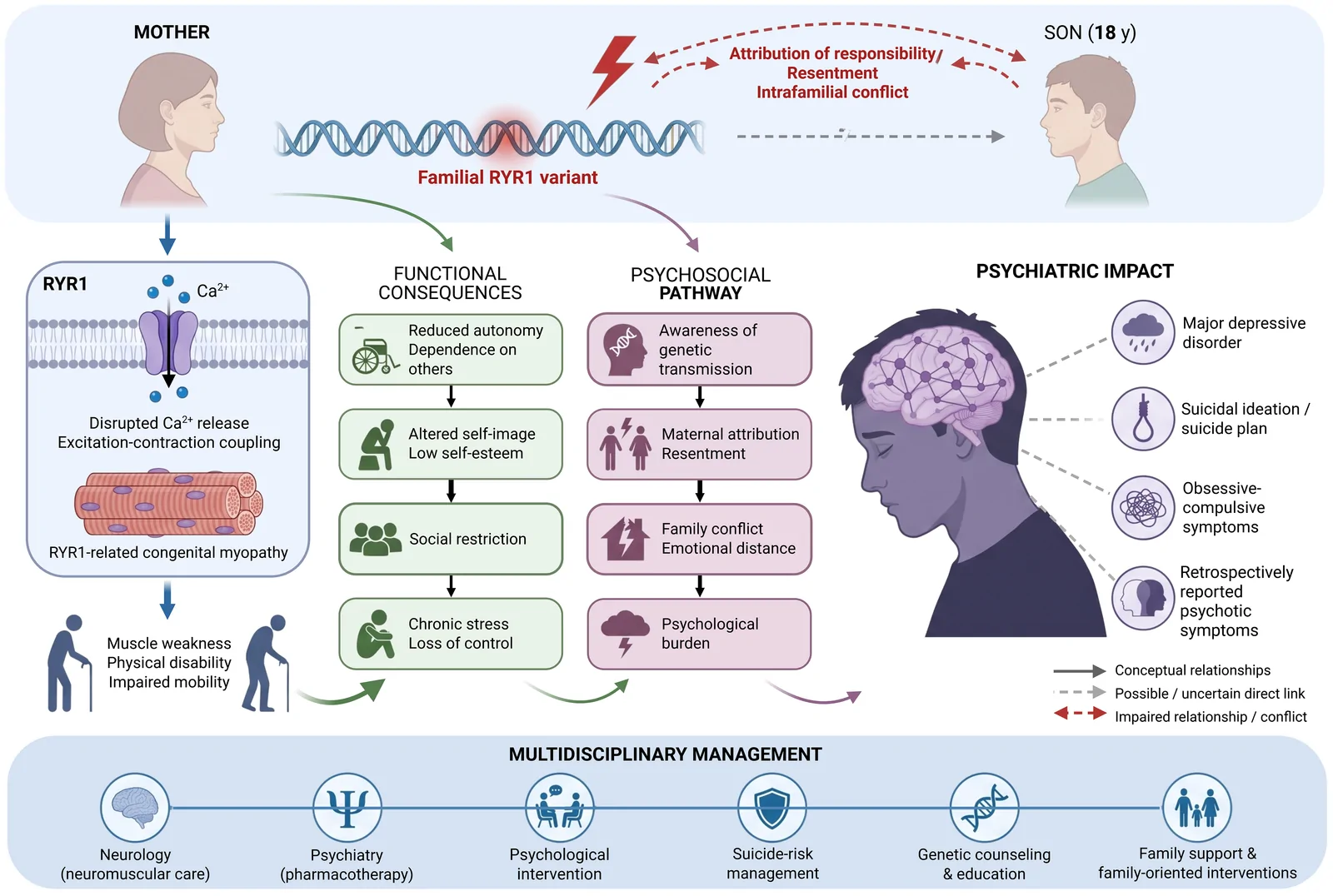
Conceptual framework of the neuromuscular and psychosocial factors considered in this case. The calcium-signaling illustration provides general biological context (30–32), and it does not represent a functional finding demonstrated for this patient’s variant. The psychosocial arrows illustrate possible relationships rather than causal effects established by the report. Connecting lines are schematic and do not establish a direct biological relationship between RYR1 dysfunction and psychiatric symptoms. Management components illustrate a multidisciplinary framework and do not confirm delivery of every intervention; completion of formal genetic counseling was not documented. Created in BioRender. Vasiliu, O. (2026). https://BioRender.com/syzlv4d.

Reports of RYR1-related myopathy describe a range of inheritance patterns, clinical courses, and neuromuscular phenotypes (33–35). Severe presentations with respiratory involvement have also been reported (36, 37). A case of congenital myopathy with a *de novo* dominant RYR1 variant was described by Jungbluth et al. (2007) in a 16-year-old girl with clinical manifestations of congenital myopathy and external ophthalmoplegia (38). In that case, mutational analysis detected a *de novo* dominant missense mutation (c.12335C > T; Ser4112Leu) affecting a highly conserved domain of the RyR1 protein (38). Other reports describe recessive RYR1-related disease, including the series reported by Wilmshurst et al. (2010) and Abath Neto et al. (2017) (39, 40). Laughlin et al. reported a patient carrying two RYR1 variants in compound heterozygosity, one pathogenic frameshift variant and one missense variant of uncertain significance (41). However, these cases do not report systematically on psychiatric manifestations, which leaves a gap in our current knowledge about the impact of these pathologies on the patients’ mental status. Two relevant sources about this dimension of healthcare include the study conducted by van Ruitenbeek et al. (2019), which assessed in 72 patients the psychological burden of the disease, and showed that substantial fatigue and impairment of quality of life exist in the RYR1-related myopathies group vs. those with malignant hyperthermia or exertional rhabdomyolysis (6). The other study explored the consequences of living with RYR1-related diseases in patients and caregivers (N = 277 participants) and identified mental-health impact as a recurrent theme in testimonials (n=19 quotations); these reports were about consequences of living with these genetic diseases, not about a causal relationship between RYR1 variants and the onset of psychiatric manifestations (7).

The relevant feature of this case lies in the association of an RYR1-related congenital myopathy, with an autosomal dominant family pattern, with a severe depressive disorder, accompanied by active suicidal ideation and obsessive-compulsive disorder, in a young patient at a critical stage of psychosocial development. The familial RYR1 p.Leu4579Arg missense variant was identified in both the patient and his clinically affected mother, supporting segregation with the neuromuscular phenotype. At the time of the original molecular investigation, several features were considered supportive of pathogenicity, although functional characterization was not available. While RYR1-related disorders are known to include malignant hyperthermia susceptibility, a variant-specific association between p.Leu4579Arg and malignant hyperthermia has not been established. Patient-specific muscle histopathology was unavailable, so a central core or centronuclear subtype cannot be assigned to him. Centronuclear histology occurs in genetically distinct myopathies, including MTM1-related X-linked myotubular myopathy (42–44).

Several features of the case are relevant to its psychiatric formulation:

1. CMs are rare conditions, and the literature provides limited descriptions of the associated psychiatric impact; in this case, the documented depressive presentation met DSM-5-TR criteria for major depressive disorder and required specific psychiatric treatment and suicide-risk management. This diagnostic formulation does not imply etiological independence from the neuromuscular disease or its psychosocial consequences;
2. Intermittent suicidal thoughts were retrospectively dated to 2019; active suicidal ideation with a plan was documented during the later psychiatric assessment. This history highlights the need for continued suicide-risk assessment without establishing the onset of syndromal depression in 2019;
3. Severe depressive episode, obsessive-compulsive disorder, and retrospectively reported psychotic symptoms add to the psychiatric burden; the diagnostic significance of the earlier psychotic symptoms remains uncertain;
4. The impact of disability on identity and relational development is reflected in significant motor limitations, partial functional dependence, and difficulties in forming interpersonal and affective-sexual relationships; these may be relevant to decreased self-esteem, social withdrawal, and the internalization of feelings of inferiority;
5. Response to a multimodal therapeutic strategy. The case highlights the need for a comprehensive approach: antidepressant (SSRI), antipsychotic augmentation, mood stabilizer (lamotrigine), and recommended psychotherapeutic interventions; the partial clinical improvement observed following multimodal treatment illustrates the feasibility of an individualized psychiatric approach in this complex clinical context; however, no conclusions regarding treatment efficacy or generalizability can be drawn from a single case. The contribution of individual interventions cannot be separated.

An additional feature of this case is the potential psychological impact of the familial transmission of the neuromuscular disorder. The identification of the same RYR1 variant in the patient and his affected mother provided a concrete framework through which the patient could attribute his illness to maternal inheritance. The variant was identified after his birth, and the available documents do not establish what the parents knew or which reproductive options were available to them. In this context, his marked resentment toward his mother and tendency to hold her responsible for transmitting the disorder may reflect the broader psychological challenges associated with living with an inherited disabling condition. Beyond the burden of physical limitations, awareness of genetic transmission may influence family relationships, illness perceptions, autonomy, and emotional adjustment, particularly during the transition to adulthood. Although a causal contribution to the patient’s depressive disorder cannot be established, this intrafamilial dimension may have represented an additional source of psychological distress and highlights the potential value of integrating genetic counseling and family-oriented psychological support into the multidisciplinary management of inherited neuromuscular disorders. Formal genetic counseling was recommended, but attendance was not documented. Also, the family-oriented psychiatric work should not be equated with formal genetic counseling.

## 4 Limitations

There are several limitations of this case report, such as the lack of structured monitoring for OCD symptoms on a validated instrument, such as the Yale-Brown Obsessive-Compulsive Scale (YBOCS), and for suicidal manifestations (such as the Beck Suicide Scale, BSS), lack of enough information about the retrospectively reported psychotic symptoms (which are only declarative and supported by the mother), and the need for more relevant data about the familial history of the CM beyond the mother’s disease. Also, the causal relationship between RYR1 CM and the psychiatric disorders could not be supported based on the current design of our report. The use of GAF, excluded from DSM-5 and DSM-5-TR, was considered useful due to its overall assessment significance, even if it is now considered of historical value (13, 45). It is acknowledged, however, that GAF cannot replace symptom-specific measures. Additional limitations include the single-case design, retrospective reconstruction of part of the psychiatric history, and inability to determine the relative contribution of individual components of the multimodal treatment to the observed improvement. A systematic family psychiatric history, confirmation of completed formal genetic counseling, and patient-specific muscle histopathology were unavailable. Comparable serial motor measurements and dates of specific functional losses were lacking, preventing assessment of the temporal relationship between physical deterioration and psychiatric worsening. Some treatment details, including the timing of gabapentin titration and the delivery and intensity of recommended psychotherapy, were also unavailable.

Directions for future research involve the longitudinal exploration of mental health status in patients diagnosed with RYR1-myopathies, using structured methods. The psychological burden of the disease should be assessed regularly in the patients and in their caregivers, too, in order to find the most appropriate measures to mitigate such burden. Case-based research in treatment-resistant depression illustrates the use of structured symptom and quality-of-life measures to guide individualized follow-up (46).

## 5 Conclusion

This case illustrates the clinically significant psychiatric burden that may accompany RYR1-related congenital myopathy and highlights the complex interaction between chronic physical disability, impaired autonomy, genetic disease awareness, family dynamics, and psychiatric morbidity. Although a direct pathophysiological relationship between RYR1 dysfunction and major depressive disorder cannot be inferred from this case, the presence of severe depression, suicidal ideation, and obsessive-compulsive disorder emphasizes the importance of systematic mental-health assessment in patients with rare disabling neuromuscular conditions. Multidisciplinary management integrating neurological and psychiatric care, psychological interventions, suicide-risk assessment, genetic counseling, and family-oriented support may be particularly valuable in addressing the complex needs of these patients. This case report does not establish an RYR1-specific psychiatric phenotype or the efficacy of an individual treatment, both of which are important directions for future research in this field.

## Data Availability

The original contributions presented in the study are included in the article/supplementary material, further inquiries can be directed to the corresponding author/s.
